# Site-Specific Impact of a Regional Hydrodynamic Injection: Computed Tomography Study during Hydrodynamic Injection Targeting the Swine Liver

**DOI:** 10.3390/pharmaceutics7030334

**Published:** 2015-09-16

**Authors:** Takeshi Yokoo, Tsutomu Kanefuji, Takeshi Suda, Kenya Kamimura, Dexi Liu, Shuji Terai

**Affiliations:** 1 Division of Gastroenterology and Hepatology, Graduate School of Medical and Dental Sciences, Niigata University, 1-757 Asahimachi-dori, Chuo-ku, Niigata, Niigata 950-8510, Japan; E-Mails: t-yokoo@med.niigata-u.ac.jp (T.Y.); kenya-k@med.niigata-u.ac.jp (K.K.); terais@med.niigata-u.ac.jp (S.T.); 2 Department of Gastroenterology & Hepatology, Uonuma Institute of Community Medicine, Niigata University, 4132 Urasa, Minami Uonuma, Niigata, 949-7392, Japan; E-Mail: kanefuji@med.niigata-u.ac.jp; 3 Department of Pharmaceutical and Biochemical Sciences, University of Georgia, College of Pharmacy, 250 W. Green street, Athens, GA 30602, USA; E-Mail: dliu@uga.edu

**Keywords:** hydrodynamic gene delivery, swine, computed tomography, interventional radiology, regional application, hemodynamics

## Abstract

A hemodynamic study of hydrodynamic gene delivery (HGD) from the tail vein in rodents has inspired a mechanism and an approach to further improve the efficacy of this procedure. However, there is no report on the hemodynamics of a regional HGD, which is an inevitable approach in large animals. Here, we report the hemodynamics of a regional hydrodynamic injection in detail based on 3D volume data and the dynamism of tissue intensity over time by using computed tomography (CT) both during and after a regional hydrodynamic injection that targeted the liver of a pig weighing 15.6 kg. Contrast medium (CM) was injected at a steady speed of 20 mL/s for 7.5 s under the temporal balloon occlusion of the hepatic vein (HV). A retrograde flow formed a wedge-shaped strong enhancement area downstream of the corresponding HV within 2.5 s, which was followed by drainage into another HV beginning from the target area and the portal vein (PV) toward a non-target area of the liver. After the injection, the CM was readily eliminated from the PV outside the target area. These data suggest that an interventional radiology approach is effective in limiting the hydrodynamic impacts in large animals at a target area and that the burden overflowing into the PV is limited. A further investigation that simultaneously evaluates gene delivery efficiency and hemodynamics using CT is needed to establish feasible parameters for a regional HGD in large animals.

## 1. Introduction

Hydrodynamic gene delivery (HGD) from the tail vein in rodents is known to be an efficient and safe procedure for *in vivo* gene delivery, especially to the liver, and it has been utilized in many laboratories worldwide since it was first reported in 1999 [1,2,3,4,5,6]. Hemodynamic and morphological impacts have been extensively studied in mice [7,8,9,10,11,12] and provide a mechanistic implication and a clue to further improving this procedure. Because HGD is considered to possess the smallest inherent risk for biological adverse events, several researchers are enthusiastically attempting to modify this procedure for large animals by using pigs [13], dogs [14], and *ex vivo* human liver segments [15] and aiming at clinical applications.

To avoid a devastating disturbance of circulation, reducing the volume is a necessary task for a large animal application of HGD. We introduced interventional radiology techniques to restrict the target area and reduce the injection volume. We call the modified procedure regional HGD, in contrast to an authentic HGD from the mouse tail vein, which is called systemic HGD. Although a highly reproducible result can be anticipated in systemic HGD when a proper combination of injection volume and speed is used based on the mouse body weight [16,17], this combination does not ensure reproducible gene delivery efficiency in regional HGD. In a hydrodynamic injection from the mouse tail vein, an injected solution simply refluxes from the hepatic vein (HV) to the portal vein (PV) through the sinusoidal space [12]. In the case of regional HGD, however, various drainage routes can be anticipated through physiological vascular connections. To establish a specific control system that can allow regional HGD to be a reproducible procedure, a thorough understanding of the hemodynamics observed in regional HGD is essential.

In this study, we analyzed hemodynamics by using computed tomography (CT) during a liver-targeted regional hydrodynamic injection in a pig and clarified a characteristic hemodynamic feature of a regional hydrodynamic injection. The implication for establishing a reproducible regional HGD was discussed.

## 2. Experimental Design

All animal experiments were conducted in full compliance with regulations and approved by the Institutional Animal Care and Use Committee at the Niigata University on 12 September 2014 (#126-169-3).

A 1-year-old female micro mini pig™ was purchased from Fuji Micra Inc. (Fujinomiya, Japan), and her body weight was 15.6 kg. She was sedated by xylazine (Bayer Holding Ltd., Osaka, Japan) and anesthetized using propofol (Nichi-Iko Pharmaceutical Co., Ltd., Toyama, Japan) and isoflurane (AbbVie Inc., Tokyo, Japan). A balloon catheter (9 Fr Optimo™, Tokai Medical Products Inc., Kasugai, Japan) was inserted from the right jugular vein through the 9 Fr sheath (Cook Japan Inc., Tokyo, Japan) and guided to a branch of the HV in the right medial lobe, which was coaxially aligned with a 0.035-inch Radifocus™ wire (Terumo Corp., Tokyo, Japan) under a fluoroscope (Clearscope 1000™, Toshiba Medical Systems Corp., Ohtawara, Japan). After occluding the branch by inflating the balloon, we expected the target volume would be 50 to 60 mL and injected 150 mL of contrast medium (Imagenil™, Guerbet Japan Co., Ltd., Tokyo, Japan) at a speed of 20 mL/s using a conventional injector for CM (PRESS PRO™, Nemoto Kyorindo Co., Ltd., Tokyo, Japan) according to our previous observations, which indicated that an injection of 2.5 times target volume in approximately 10 s gave rise to the fine gene delivery efficiency in a regional HGD targeting the swine liver [18]. CT scans were performed before, during and immediately after the injection (Alexion™ TSX-032A, Toshiba Medical Systems Corp., Ohtawara, Japan). The entire liver was scanned with a 1-mm thickness. In addition, a single-level CT scan was carried out throughout the injection, in which the same single axial tomogram was repeatedly captured every 0.5 s. The balloon was not deflated until all CT studies were finished. Images of the CT scans were analyzed using OsiriX Lite (version 6.5.2, Pixmeo Inc., Bernex, Swizerland).

## 3. Results and Discussion

### 3.1. Venography of the Hepatic Vein in the Right Medial Lobe

**Figure 1:**
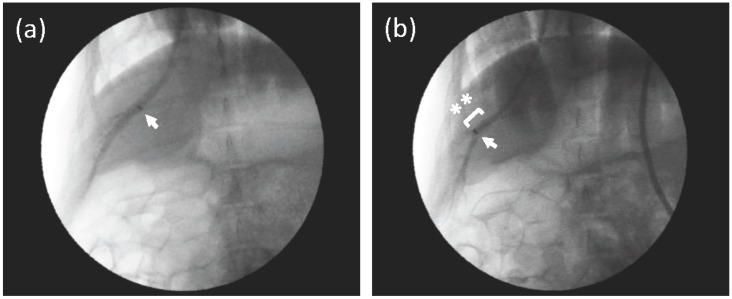
Venography of the hepatic vein (HV) in the right medial lobe. (**a**) A small amount of contrast medium (CM) was injected from the proximal part of the HV, where a catheter tip was placed (arrow) to obtain a bird’s eye view of the HV in the right medial lobe. The HV was not occluded. (**b**) A fluoroscopic image of the injection of CM from the middle part of the HV (arrow, catheter tip) with a balloon occlusion (asterisks). CM was gently injected into the HV, and the peripheral branches were enhanced. The diameter of the HV, where the tip was placed in Figure 1b, was approximately 8 mm.

A catheter was inserted into the HV of the right medial lobe coaxially following a guide wire (Radifocus™). Figure 1a shows a bird’s eye view of the HV and the location of a catheter tip with an injection of small amount of contrast medium (CM). As shown in Figure 1b, the tip was inserted to a deeper point, and a balloon was then inflated to occlude the HV. The diameter of the vessel, where the tip was placed, was approximately 8 mm, as shown in Figure 1b. The volume of the peripheral area could not be estimated clearly in the venography.

### 3.2. Single-Level Computed Tomography

**Figure 2:**
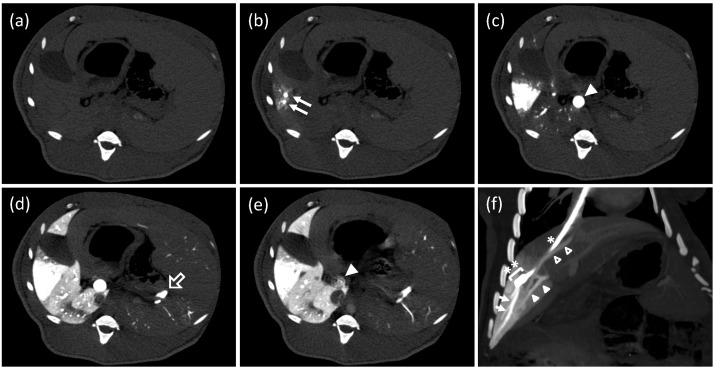
Computed tomography (CT) study during a hydrodynamic injection from a catheter, which was placed at a branch of the hepatic vein. (**a**–**e**) CT images at the same axial level were repeatedly obtained with a 0.5 s interval before, during, and after a regional hydrodynamic injection of contrast medium at a constant speed of 20 mL/s in 7.5 s. Each image was taken before the injection, (**a**); 1 s, (**b**); 2.5 s, (**c**); 7.5 s, (**d**); and 12 s, (**e**); after the injection. Closed arrows: hepatic vein (HV) branches in the target area; closed arrowhead: main trunk of the portal vein (PV); open arrow: splenic vein. (**f**) An oblique multi-planar reconstruction image immediately after the end of a hydrodynamic injection before removing a balloon occlusion. The coronal image was reconstituted with a 17.7-mm thickness. A catheter (*) with a balloon (**) was placed in the branch of hepatic vein via the right jugular vein. Arrows: periphery of the target HV; closed arrowheads: portal vein of the target area; open arrowheads: another hepatic vein beginning from the inside of the target area.

A single-level CT scan revealed that the HV in the target area was filled with CM immediately after initiating the injection. At the same time, the corresponding parenchyma became enhanced in a wedged shape (Figure 2b). CM passing through the sinusoids flowed back to the main trunk of the PV and then to the peripheral PV branches of a non-target area of the liver at 2.5 s after starting the injection (Figure 2c). In Figure 2d, CM in the peripheral PV branches flowed into the sinusoids in the non-target area, and parenchyma was enhanced. Although CM reached the splenic vein (SpV), splenic parenchyma was not enhanced. Immediately after the injection finished, the anterograde flow in the main trunk of the PV was quickly restored, and CM was fairly washed out (Figure 2e). No shunt vessels were observed.

**Figure 3:**
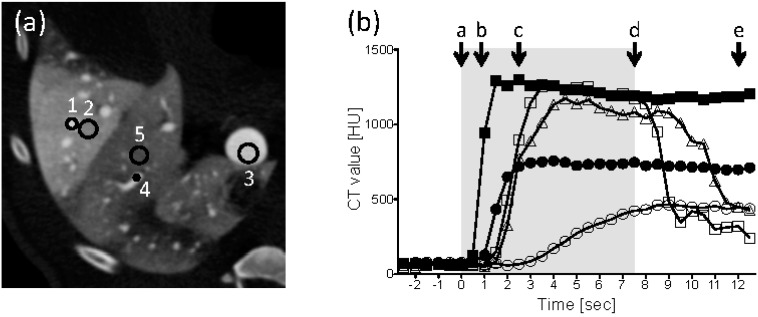
Tissue intensity during a regional hydrodynamic injection. (**a**) The image from Figure 2d is shown with a different window width and center to represent the vessels. The regions of interest that were used to measure the CT values were the HV (target), liver parenchyma (target), the main trunk of the PV, a peripheral PV branch (non-target), and liver parenchyma (non-target), which were marked 1 to 5, respectively. (**b**) Time intensity curves. A period of the injection is indicated by a gray box. The time points corresponding to the Figure 2a–e are represented by a–e. Closed squares: HV (target); closed circles: parenchyma (target); open squares: main trunk of the PV; open triangles: peripheral PV (non-target); open circles: parenchyma (non-target).

Figure 3a,b show the five points of the HV (target), liver parenchyma (target), the main trunk of the PV, a peripheral PV branch (non-target), and liver parenchyma (non-target), where CT values were measured and the values at each point that were obtained every 0.5 s, respectively. Immediately after the injection started, the CT value of the target HV sharply increased, reached a peak level and was maintained at least 5 s after the end of the injection. Similar time-intensity curves were recorded at the target parenchyma, the main trunk of the PV, and a peripheral PV branch in the non-target region, whereas the peak intensities ranged from slightly below to almost 50% of the value found in the HV. In contrast, the CT value of the non-target liver parenchyma increased gently and peaked at approximately one third of that observed in the target HV. As the injection finished, the CT values of the main trunk and peripheral PV branch quickly decreased.

### 3.3. Conventional Enhanced Computed Tomography

**Figure 4:**
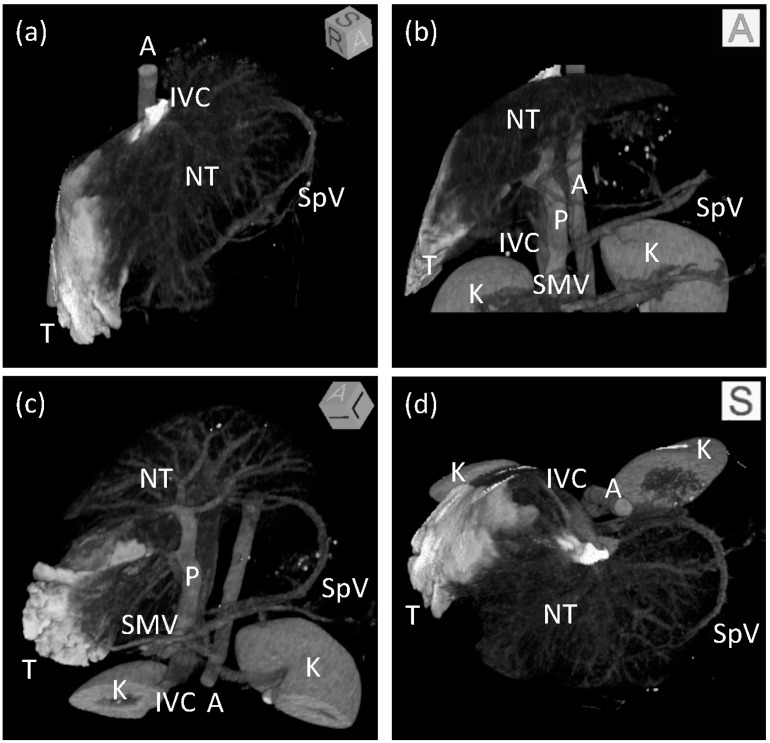
Three-dimensional images of the liver after regional hydrodynamic injection. Maximum intensity projection images after volume rendering viewing from the (**a**) right anterior superior, (**b**) anterior, (**c**) left anterior inferior, and (**d**) superior directions. The target area is shown in white. Branches of the hepatic and portal vein are visible in the non-target area of the liver. Boxes at the upper right corner of each image show an angle of 3D images. T: target area; NT: non-target area; IVC: inferior vena cava; P: portal vein; SpV: splenic vein; SMV: superior mesenteric vein; A: aorta, K: kidney.

A conventional enhanced computed tomography of the upper abdomen, including the whole liver, was performed approximately 1 min after the end of the single-level CT study with the balloon inflated. At that time, CM had already reached the aorta and enhanced abdominal organs through the visceral arteries. The main trunk of the PV was enhanced again by an anterograde flow from the SpV and superior mesenteric vein. An oblique multi-planar reconstruction image is shown in Figure 2f, which shows the tip of the balloon catheter, the target area with a wedge-shaped enhancement, another HV beginning from the target area, and branches of the PV corresponding to the target area. The volume of the target area was 52.5 mL, which was equivalent to 16.6% of the whole liver (Figure 4).

### 3.4. Discussion

HGD is a promising human gene therapy method and may incur fewer adverse events such as carcinogenesis and allergic reaction [19,20,21]; further, the hepatocytes can be targeted up to 40% of the liver at once. We previously unveiled the hemodynamics of a hydrodynamic injection through the mouse tail vein without any surgical procedure and provided basic insight to further improve the efficacy of this technology [12]. In large animals, however, HGD must be applied regionally by targeting an organ or part of an organ to preserve systemic circulation. The hemodynamics of HGD must be investigated in large animals to adjust the injection parameters used in this technology to allow for a regional application. This is the first report to show the hemodynamics of a regional hydrodynamic injection in detail by targeting the pig liver and using CT, which provided 3D data on the volume and dynamism of tissue intensity over time.

The prominent hemodynamic difference between systemic and regional applications of hydrodynamic injection is the different drainage routes of the injected solution. Although the main drainage route is the portal vein and the drain is readily washed out by the natural blood flow shortly after the end of the injection in both the systemic and regional approaches, the flow direction of the drain differs between the systemic and regional hydrodynamic injections. In a hydrodynamic injection from the mouse tail vein, CM traveled from the inferior vena cava (IVC) and HV to the PV and then toward the other side of the liver, though always under resistant pressure from the natural blood stream [12]. In contrast, in a regional hydrodynamic injection, CM passed the HV (target), parenchyma (target), PV (main trunk), PV (non-target), and parenchyma (non-target) in this order, as indicated in the time when the tissue intensity peaked in CT (Figure 3b). CM flowed into the non-target area of the liver in a natural flow direction through the connections of the PVs between the target and non-target areas in a regional hydrodynamic injection. Once the injected solution reached the portal branch, which is “a critical point” where PV branches connect target and non-target areas, the solution was actively drained into systemic circulation in the same direction as the natural blood stream.

In our condition, the target area was 52.5 mL (16.6%), which suggests that 100 mL or more CM was drained from the target area. Even with the larger amount of CM, the intensity at the non-target area could be lower than that at the target area because the volume difference between the non-target and target areas outstripped the different amounts of CM. On the other hand, many other factors may be involved in the slower increase in intensity observed at the non-target parenchyma compared with the target parenchyma (Figure 3b). A maximum of 20 mL of CM made it into the target (16.6% of the liver) or non-target (83.4% of the liver) every second, which suggests that the flow speed of CM in the non-target would be one fifth or less of that found in the target. Bidirectional flow at the critical point would divide the flow between the non-target liver parenchyma and the superior mesenteric vein. The influx of blood without CM via the hepatic artery can be another factor that deteriorates the enhancement due to CM in the non-target area, where there is no outflow block compared with the balloon occlusion at the hepatic vein in the target area. Furthermore, the flow volume of CM may vary in the drain from the target over the course of the injection due to the dislocation of CM from the intravascular to extravascular space. If that were the case, a sustained injection after the injected solution reaches “the critical point” would be effective for gene delivery; otherwise, the injection should be stopped shortly after the solution reaches “the critical point” because it is well known that hydrodynamic injection from the PV does not work well without the outflow block at the IVC unless the injection volume is sufficiently large to induce systemic circulation disturbance in mice. The relation between injection volume and gene delivery efficiency in regional HGD must be thoroughly investigated along with hemodynamic study.

In regional HGD, the proper combination of injection speed and volume cannot be defined prior to starting the injection in each case because of both the uncertain target volume and occasional drainage. The inconsistent previous reports in terms of gene delivery efficiency among target and non-target liver lobes strongly indicated the appearance of occasional drainages during a regional HGD [22,23]. Compared with the backflow, which has a steady direction in a hydrodynamic injection from the mouse tail vein, bidirectional drainage in a regional hydrodynamic injection can change easily on a case-by-case basis due to faint difference in the anatomical structures and/or injection conditions. Direct drainage into the non-occluded hepatic vein beginning from the target area is also unspecifiable before starting an actual injection. This report clearly showed that the variable drainage route and direction observed in a regional hydrodynamic injection could explain the fact that a specific injection control system is required to make a regional HGD reproducible. To control the injection, the liver expansion rate is one of the most promising determinants [12,18]. The target volume should be evaluated over a course of the injection to define suitable injection parameters. It is very difficult, however, to assign the target area prior to an actual injection. Ming Ching reported that the diameter of a branch of splenic artery was positively correlated with the volume of the corresponding parenchyma of the spleen [24]. Consistently, the diameter of the target HV may be correlated with the target volume. To verify this hypothesis, the relationship between the HV diameter and the target volume must be investigated further by using CT scanning, especially in a single-level study.

## 4. Conclusions

The hemodynamics of a regional hydrodynamic injection was evaluated using single-level and conventional enhanced CT studies in a pig. A restoration of the natural portal flow soon after cessation of the injection was confirmed, as observed following a hydrodynamic injection from the mouse tail vein. Although a substantial amount of the injected solution was drained into the non-target liver through both the PVs and another HV beginning from the target area, an obviously higher intensity was generated downstream of the target HV, which suggests that a hydrodynamic impact can be localized by employing interventional radiology techniques. A further study that simultaneously evaluates gene delivery efficiency and hemodynamics can provide us with clues for establishing feasible efficiency and safety parameters for regional HGD in the clinic.
